# Down-regulation of colon mucin production induced by *Eimeria pragensis* infection in mice

**DOI:** 10.3389/fpara.2025.1621486

**Published:** 2025-06-24

**Authors:** Yulia Dwi Setia, Mio Kokubo-Tanaka, Ryusei Tanaka, Akemi Yoshida, Eiji Nagayasu, Parnian Ahmadi, Ayako Yoshida, Haruhiko Maruyama

**Affiliations:** ^1^ Division of Parasitology, Department of Infectious Disease, Faculty of Medicine, University of Miyazaki, Miyazaki, Japan; ^2^ Department of Clinical Parasitology, Faculty of Medicine, Universitas Brawijaya, Malang, Indonesia; ^3^ Laboratory of Genomics, Frontier Science Research Center, University of Miyazaki, Miyazaki, Japan; ^4^ Center of Animal Disease Control, University of Miyazaki, Miyazaki, Japan; ^5^ Laboratory of Veterinary Parasitic Diseases, Department of Veterinary Sciences, Faculty of Agriculture, University of Miyazaki, Miyazaki, Japan; ^6^ Human and Molecular Genetic Research Department, Virginia Commonwealth University, Richmond, VA, United States

**Keywords:** *Eimeria pragensis*, goblet cell dysfunction, colitis, mucin down-regulation, mouse model

## Abstract

**Introduction:**

*Eimeria pragensis*, an intestinal protozoa infecting mice, induces colitis and reduces goblet cell numbers in the large intestine. In the present study, we investigated the pathogenesis and the mechanisms underlying goblet cell down-regulation in the early phase of infection.

**Methods:**

Male C57BL/6 mice were orally infected with 300 oocysts. Fecal oocyst shedding and body weight were monitored daily. Colon tissues were collected at 3, 8, and 13 days post-infection (dpi) to assess pathological changes. Parasite burden was assessed by histological analysis (H&E staining) and qPCR targeting 5S rRNA. Goblet cells were visualized using PAS-Alcian Blue staining and Muc2 immunohistochemistry. To elucidate mechanisms of goblet cell dysfunction, we performed RNA sequencing of large intestine tissue to examine host as well as parasite transcriptomes.

**Results:**

Fecal oocyst excretion peaked at 8–9 dpi. Body weight decreased from 6 to 11 dpi, with recovery after 12 dpi. Maximal parasite accumulation in the proximal colon was observed at 8 dpi in histological examination as well as qPCR. Colon length was significantly shortened at 3 dpi. Goblet cell area significantly reduced at 8 dpi (p < 0.05). RNA sequencing of infected large intestines revealed that *E. pragensis* produced enzymes that were known to degrade mucin and tight junctions, and proteins that could activate the Notch–Hes1 signaling pathway. As for host responses, genes associated with Th1-type inflammation, epithelial barrier disruption, and immune regulation were up-regulated as early as 3 dpi.

**Discussion:**

Our findings suggested that *E. pragensis* infection induces a mucosal barrier dysfunction in the early phase of the infection, which possibly causes the tissue invasion of bacteria in the large intestine. Th1-type inflammatory response, thus induced, reduces goblet cell numbers and mucin production. This model provides valuable insight into the mechanisms of mucosal barrier disruption during protozoan infection.

## 1 Introduction

Species of *Eimeria* are protozoan parasites belonging to Coccidiasina (Coccidia), a group of obligate intracellular parasites of wild and domestic animals. All members of the Coccidia replicate within the intestines of a definitive host progressing through sequential rounds of asexual (schizogony) and sexual (gametogony) reproduction, culminating in the production of oocysts that are shed into the environment with the feces (Blake and Tomley, 2014; Burrell et al., 2020). Coccidians of the family Eimeriidae, such as species of *Eimeria* and *Cystoisospora*, are monoxenes; their development is restricted to a single host where they replicate rapidly to reach high numbers in the intestine causing acute enteritis of varying severity (Burrell et al., 2020).

*Eimeria pragensis* is native to mice and infects colon epithelial cells (Yunus et al., 2005b). At the peak of the infection, significant tissue damage and inflammation take place, and the integrity of the Muc2 layer is compromised due to the depletion of goblet cells (Yunus et al., 2005a; Uemura et al., 2022). Loss of mucin layer exposes underlying epithelial cells to luminal bacteria and toxins (Kim and Ho, 2010; Chapman, 2014; Pelaseyed et al., 2014; Lu et al., 2021), contributing to the ‘leaky gut’ phenomenon (Chapman, 2014; Borisova et al., 2020). This breach not only exacerbates inflammation but also facilitates secondary bacterial infections, further damaging the intestinal lining (Kim and Ho, 2010; Chapman, 2014; Pelaseyed et al., 2014; Lu et al., 2021; Uemura et al., 2022). Thus, infected animals suffer from diarrhea and weight loss (Siswandi et al., 2018; Günay-Esiyok and Gupta, 2024). It has been demonstrated that Muc2 depletion plays a central role in the pathogenesis of *E. pragensis* (Dkhil et al., 2013; Sun et al., 2024). However, little is known as to how mucosal inflammation and goblet cell downregulation are triggered.

In this study, we aim to investigate how *E. pragensis* contributes to goblet cell depletion and colon mucin downregulation in the colon of mice. We combined histopathological evaluation, molecular profiling and dual RNA-seq approaches to uncover the dynamic host-parasite interactions that shape mucosal immune responses and barrier dysfunction during infection.

## 2 Materials and methods

### 2.1 Parasites and animals

*E. pragensis* was obtained from the Institute for Animal Health, Compton Laboratory, UK, and maintained in the Parasitology laboratory University of Miyazaki through periodic propagation of sporulated oocysts every 3 months. The propagation process involves several steps. (First, fecal material was collected from infected mice while still fresh to maximize oocyst viability. Oocysts were then isolated using a flotation method with a sugar solution of specific gravity 1.2, and contaminants such as debris and bacteria were removed using a 30% sodium hypochlorite solution. The isolated oocysts were incubated under optimal environmental conditions at 27 °C with a relative humidity of 80–90%. Sporulation was induced by incubating the oocysts in a 2.5% potassium dichromate solution at 27 °C for 4–5 days, until more than 80% of the oocysts were sporulated. After sequential washing with distilled water, the sporulated oocysts were stored at 4 °C until further use. For each propagation cycle, 300 sporulated oocysts were orally inoculated into mice to maintain the parasite strain (Siswandi et al., 2019).

Male C57BL/6 mice were purchased from Japan SLC, Inc. and housed in the Animal Facility of Parasitology Laboratory University of Miyazaki, with continuous access to food and water at room temperature with natural humidity levels. Seven to eight-week-old mice were orally inoculated with 300 sporulated oocysts of *E. pragensis*, suspended in 200-300 µl of distilled water using an oral gavage tube (Siswandi et al., 2019). Infection was assessed via fecal examination. At the same time, mice were measured for body weight daily. This experiment was approved by the Ethical Committee of the University of Miyazaki under approval number 2022-034-2.

The dosage of 300 sporulated oocysts was adopted from the infection protocol established by Siswandi et al. (2019), who used the same dose to reliably induce infection in 7–8-week-old male C57BL/6 mice weighing 20–25 grams. This age group represents immunologically mature and healthy animals suitable for gastrointestinal infection studies. The single oral dose was chosen to ensure consistent parasite establishment while minimizing potential adverse effects. No repeated dosing was done in this study.

The number of independent experiments varied depending on the assay. Fecal oocyst counts were measured in five animals in one experiment, following protocols established in our previous publications. Body weight changes and RNA-seq were assessed in one experiment (n = 3 per group). Colon length, histology, and qPCR data were obtained from five independent infection experiments. PAS-Alcian Blue and immunohistochemistry were each conducted in two experiments. Unless otherwise noted, each group included three biological replicates.

### 2.2 Fecal examination

The McMaster method was carried out daily to assess the oocyst count per gram. Two grams of fecal material were measured and mixed with 60 ml of 40% (w/v) NaCl solution. Subsequently, the oocyst count was conducted using a McMaster counting chamber under a microscope (Yunus et al., 2005b). Fecal samples from five mice were used as a biological replicate. Feces from the uninfected control group were also examined to ensure the absence of contamination.

### 2.3 Histological analysis

Mice infected with *E. pragensis* were euthanized at three time points: 3 dpi, 8 dpi, and 13 dpi. The cecum and colon were excised and divided into proximal, median, and distal segments. For regional parasite distribution analysis, the entire cecum and colon were longitudinally divided into six equal-length sections from proximal to distal before histological processing. These samples were fixed in 10% formalin for 48 hours, dehydrated in a sequential alcohol gradient using a rotary machine, and embedded in paraffin. Then the tissues were sectioned into 4 µm-thin slices and stained with hematoxylin and eosin (H&E).

Histopathological evaluation was performed using a scoring system modified from Qing et al. (2024), based on three parameters: inflammatory cell infiltration, mucosal hyperplasia, and the percentage of epithelial area invaded by parasites. Inflammatory infiltration was graded as follows: normal (score 0), infiltration into the lamina propria (score 1), infiltration into the submucosa (score 2), and infiltration throughout the entire intestinal wall (score 3). Mucosal hyperplasia was scored as normal (score 0), 1.5–2 times the normal thickness (score 1), threefold thickness (score 2), and fourfold thickness (score 3). Parasite invasion was scored according to the percentage of the epithelial area containing *E. pragensis* stages: 0% (score 0), 1–30% (score 1), 31–70% (score 2), and >71% (score 3). The total histopathology score was calculated by summing the three parameters, with a maximum possible score of 9 per section.

For Periodic Acid Schiff-Alcian Blue staining, the ab245876 Alcian Blue PAS Stain Kit (Abcam ^©^) was used according to the manufacturer’s protocol. Goblet cell areas in 30 crypts were quantified using ImageJ software (Schneider et al., 2012).

For Muc2 immunohistochemistry, paraffin-embedded 3 dpi, 8 dpi, and 13 dpi tissue sections underwent deparaffinization and rehydration via sequential immersion in xylene and graded alcohol solutions. Antigen retrieval was achieved by subjecting tissue sections to heat-induced treatment at 120°C for 15 minutes using citrate buffer in an autoclave machine. Endogenous peroxidase activity was neutralized, and non-specific binding sites were blocked with goat serum. Following overnight incubation at room temperature in a moist chamber with Invitrogen Muc2 Polyclonal antibody (#PA5-21329) at a 2 μg/mL concentration, tissue sections were washed and subsequently incubated with goat anti-rabbit HRP-conjugated secondary antibody (Abcam, ab6721) at 0.5 μg/mL. Visualization of target proteins was facilitated by incubation with 3,3’-diaminobenzidine (DAB) substrate for 10 minutes, followed by counterstaining with hematoxylin for 15 seconds to visualize cell nuclei. Dehydration was achieved through graded alcohol solutions, clearing in xylene, and mounting on slides with Malinol^©^, enabling microscopic observation. Muc2 areas in 30 crypts were quantified using ImageJ software (Schneider et al., 2012).

### 2.4 Parasite DNA quantification

Parasite DNA in the intestinal mucosa was quantified in qPCR of 5S rDNA. Intestinal tissues were homogenized using a tissue homogenizer, and DNA was extracted using the DNeasy Blood and Tissue Kit (Qiagen, Cat. No. 69504). The primers utilized in this study were for *Eimeria* 5SrRNA (Forward: TCATCACCCAAAGGGATT; Reverse: TTCATACTGCGTCTAATGCAC) (Blake et al., 2006). Extracted DNA samples were amplified using Takara TB Green ^®^ Premix EX Taq TM II (Tli RNaseH Plus) on the ABQuantstudio 5 platform. The qPCR cycling condition included an initial denaturation at 95°C for 30 seconds, followed by 40 cycles of 95°C for 5 seconds and 60°C for 34 seconds. Melt curve analysis was carried out with the following steps: 95°C for 15 seconds, 60°C for 1 minute and 95°C for 15 seconds. The cycle threshold (CT) values were converted to be DNA concentration based on a standard curve generated from serial dilutions using oocyst DNA (Kubista et al., 2006).

### 2.5 RNA extraction and sequencing

Colon tissues were collected from uninfected and infected mice at 3 dpi, 8 dpi, and 13 dpi, with three biological replicates per group (n = 3). Total RNA was extracted using the TRIzol™ reagent (Invitrogen, USA) according to the manufacturer’s instructions. RNA quality and integrity were assessed using an Agilent Bioanalyzer 2100 system, and only samples with RNA Integrity Number (RIN) > 7 and sufficient concentration were selected for sequencing. Qualified RNA samples were sent to Rhelixa Inc. (Tokyo, Japan) for library construction and high-throughput sequencing. Libraries were prepared using a stranded mRNA-seq protocol and sequenced on the Illumina NovaSeq 6000 platform, producing paired-end 150 bp reads (Kyunai et al., 2023).

### 2.6 Host transcripts analysis

Initial quality control of raw reads was conducted using FastQC (v0.11.9), and summary reports were generated with MultiQC (Ewels et al., 2016). Adapter trimming and removal of low-quality reads were performed using Trimmomatic v0.39 (Bolger et al., 2014). Clean reads were aligned to the *Mus musculus* reference genome (GRCm39) using the HISAT2 v2.2.1 (Kim et al., 2019). Gene-level quantification was carried out using featureCounts v2.0.6, and a raw count matrix was obtained (Liao et al., 2014). Raw count data were normalized using the transcripts per million (TPM) method.

Differential expression analysis was performed using the edgeR package in R. Genes with an adjusted p-value (false discovery rate < 0.05) and an absolute log2 fold-change > 1 were considered significantly differentially expressed. Principal component analysis (PCA) was applied to normalized counts to assess sample clustering across the time points. A heat map of differentially expressed genes (DEGs) was generated based on the TPM-normalized data to visualize expression patterns across all samples (Robinson et al., 2009).

### 2.7 Parasite transcripts analysis

RNA-seq reads above described were used for the analysis of *E. pragensis* transcripts. Due to the unavailability of a reference genome for *E. pragensis*, we used *E. falciformis* reference genome (ToxoDB-68_EfalciformisBayerHaberkorn1970), given its phylogenetic proximity of *E. pragensis* to *E. falciformis* (Ehret et al., 2017; Westermann et al., 2017). Trimmed reads were aligned to the *E. falciformis* reference genome, using the STAR aligner (v2.7.10b) (Dobin et al., 2013), with genome indices generated from the corresponding FASTA and GTF annotation files. The output consisted of BAM files sorted by genomic coordinates. Only RNA-seq data from 8 dpi could be used for downstream analysis, as the number of reads assigned to *E. falciformis* at other time points was insufficient (<10,000 reads; see **Supplementary Table S1**).

Gene-level read counts were obtained using featureCounts from the Subread package, with the GTF file as the reference. The paired-end mode was used in accordance with the original sequencing strategy. For normalization and identification of highly expressed genes, we employed the DESeq2 package in R. The raw count matrix was filtered to retain only the three 8 dpi samples. DESeq2’s internal normalization method was applied, and the average normalized expression was calculated across replicates for each gene. The top 100 genes with the highest mean normalized expression were extracted for further analysis.

### 2.8 RT-qPCR

The RNA was converted into cDNA employing the BIO-RAD iScriptTM Advanced cDNA Synthesis Kit following the manufacturer’s guidelines. The primers utilized in this study included STAT-1 (Li et al., 2020), Tbx21 (Tang et al., 2020), and Il12b (Bhattacharyya et al., 2010), with their respective sequences provided in **Supplementary Table 4**. The cDNA samples were amplified using Takara TB Green ^®^ Premix EX Taq TM II (Tli RNaseH Plus) and the ABQuantstudio 5. The qPCR cycling condition included an initial denaturation at 95°C for 30 seconds, followed by 40 cycles of 95°C for 5 seconds and 60°C for 34 seconds. Melt curve analysis was carried out with the following steps: 95°C for 15 seconds, 60°C for 1 minute and 95°C for 15 seconds. The cycle threshold (CT) values were analyzed using the Livak method, which involves relative quantification compared to actin, serving as the internal control or housekeeping gene (Leon-Coria et al., 2020).

### 2.9 Statistical analysis

Data were visualized using GraphPad Prism software. Statistical significance was evaluated using the one-way ANOVA test. When the normality and homogeneity test was not passed, we used Kruskal-Wallis’s test, with p-values less than 0.05, which was considered significant (Ghasemi and Zahediasl, 2012).

## 3 Results

### 3.1 Time course of Infection

**Figure 1:**
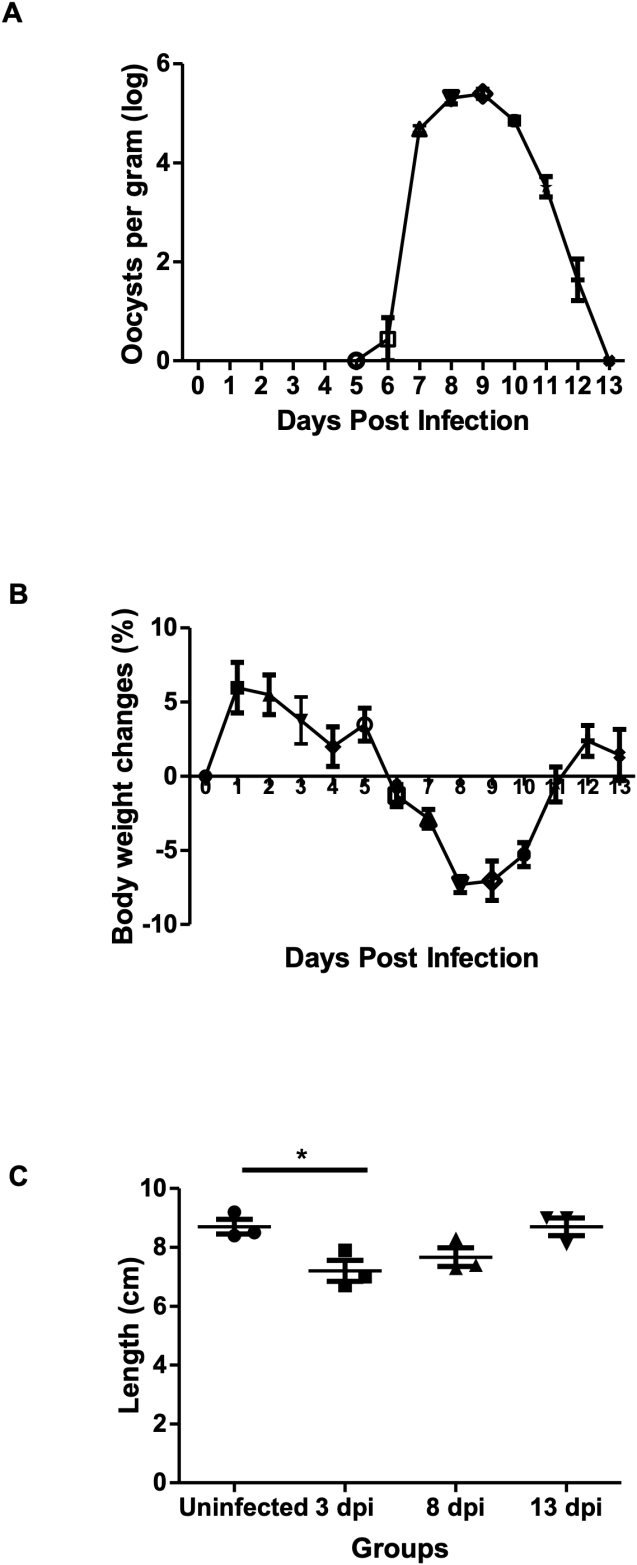
Effects of *E*. *pragensis* infection on oocyst shedding, body weight, and colon length in mice. **(A)** Oocyst excretion per gram feces measured by McMaster method (log scale). **(B)** Changes in mouse body weight compared to day 0. **(C)** Colon length measurements in uninfected and infected mice at 3, 8, and 13 dpi. Statistical significance is indicated by * (p < 0.05).

Oocysts were detected in the feces from 6 dpi, peaked at 8–9 dpi, and ceased by 13 dpi. A significant decrease in mice body weight was observed starting at 6 dpi (-1.31 ± 1.28%) and continuing until 11 dpi (-0.55 ± 2.03%) with the highest reduction peak at 8 dpi (-7.30 ± 0.94%). Colon length was significantly shorter than in uninfected controls at 3 dpi (3 dpi: 7.20 ± 0.62cm, uninfected control: 8.70 ± 0.62cm) (**Figure 1**).

**Figure 2:**
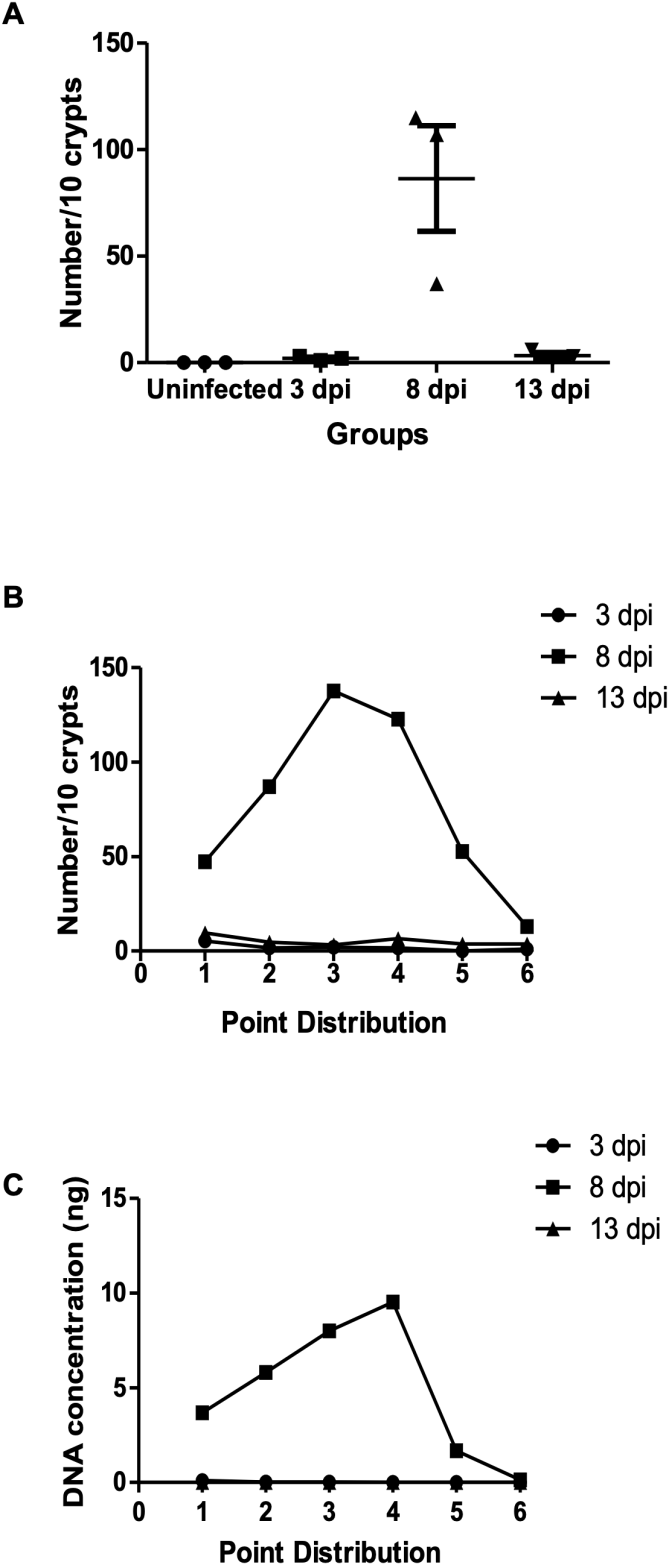
Parasite burden in intestinal tissue following *E*. *pragensis* infection. **(A)** Parasite intraepithelial count. Total number of parasites per 10 crypts at 3, 8, and 13 dpi. **(B)** Distribution of parasite counts across different regions of the cecum and colon, based on microscopic examination of H&E-stained sections. Parasites were counted per 10 crypts at 3, 8, and 13 dpi. **(C)** Distribution of parasite burden across the cecum and colon based on parasite DNA concentration (5S rRNA) measured at 3, 8, and 13 dpi.

Parasite density in colon tissue was assessed using both histology and quantitative PCR. Cecum and colon tissues were sampled at 3, 8, and 13 dpi, and each sample were divided into six sections from proximal to distal. Histological analysis revealed minimal parasite presence at 3 dpi and 13 dpi, with no significant differences across tissue sections. At 8 dpi, various developmental stages of *E. pragensis* were detectable (**Figure 2A**), with sections 3 and 4, corresponding to the proximal-median region of the colon, exhibiting the highest parasite density (**Figure 2B**). In accordance with the histology, qPCR showed large amount of parasite DNA at 8 dpi, but only minimal amount at 3 and 13 dpi (**Figure 2C**).

**Figure 3:**
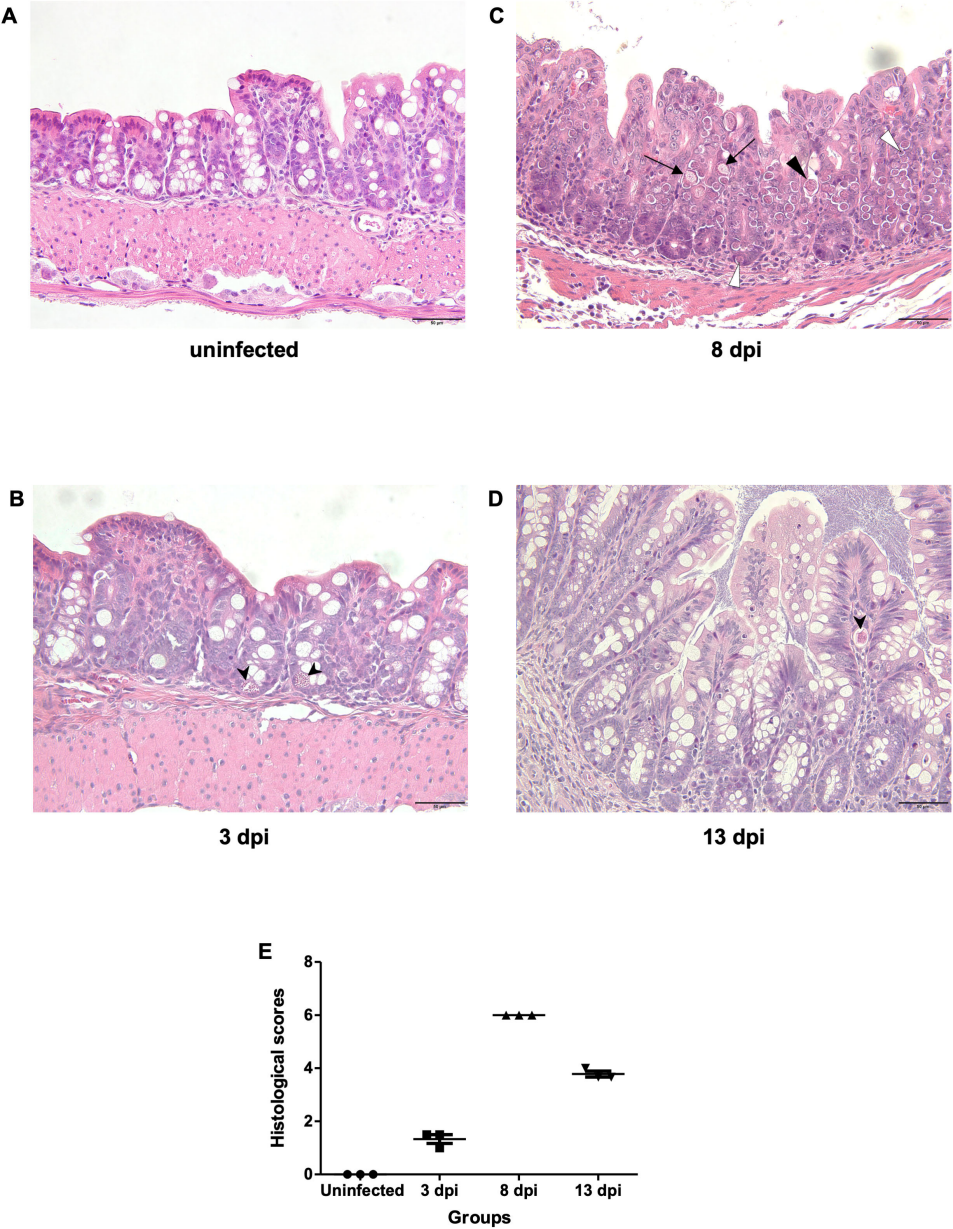
Histopathological features of the large intestine during *E*. *pragensis* infection. **(A)** Uninfected mouse, **(B)** At 3 dpi, a schizont (black arrowhead) is observed within the epithelial cells. **(C)** At 8 dpi, multiple developmental stages of *E*. *pragensis* are present, including developing oocysts (black triangle), early gametocytes (white triangle), and late gametocytes (black arrow). **(D)** At 13 dpi, developing oocysts (black arrowhead) are still visible within the epithelium, although less frequently. All images are hematoxylin and eosin (H&E) stained. Scale bars: 50 μm. **(E)** Histopathological scores of colonic tissue during infection.

Based on H&E staining, at 3 dpi, only schizont stages were observed in a small number of epithelial cells. At 8 dpi, multiple developmental stages of *E. pragensis* were detected, including gametocytes and developing oocysts located intracellularly within intestinal epithelium. At 13 dpi, a few developing oocysts were still observed (**Figures 3A-D**). Histopathological scoring revealed a significant increase in tissue damage in infected mice compared to the uninfected group. The uninfected group showed a consistent score of 0, indicating no inflammation or parasite invasion. At 3 dpi, the average score slightly increased (1.33 ± 0.29), reflecting mild inflammation and low-level parasite presence. The highest histological scores were observed at 8 dpi (6.00 ± 0.00), corresponding to extensive inflammatory infiltration, mucosal hyperplasia, and high parasite burden. At 13 dpi, the scores remained elevated (3.78 ± 0.19) but were lower than at 8 dpi, suggesting a partial resolution of tissue damage (**Figure 3E**).

### 3.2 Goblet cell dynamics during *E. pragensis* infection

**Figure 4:**
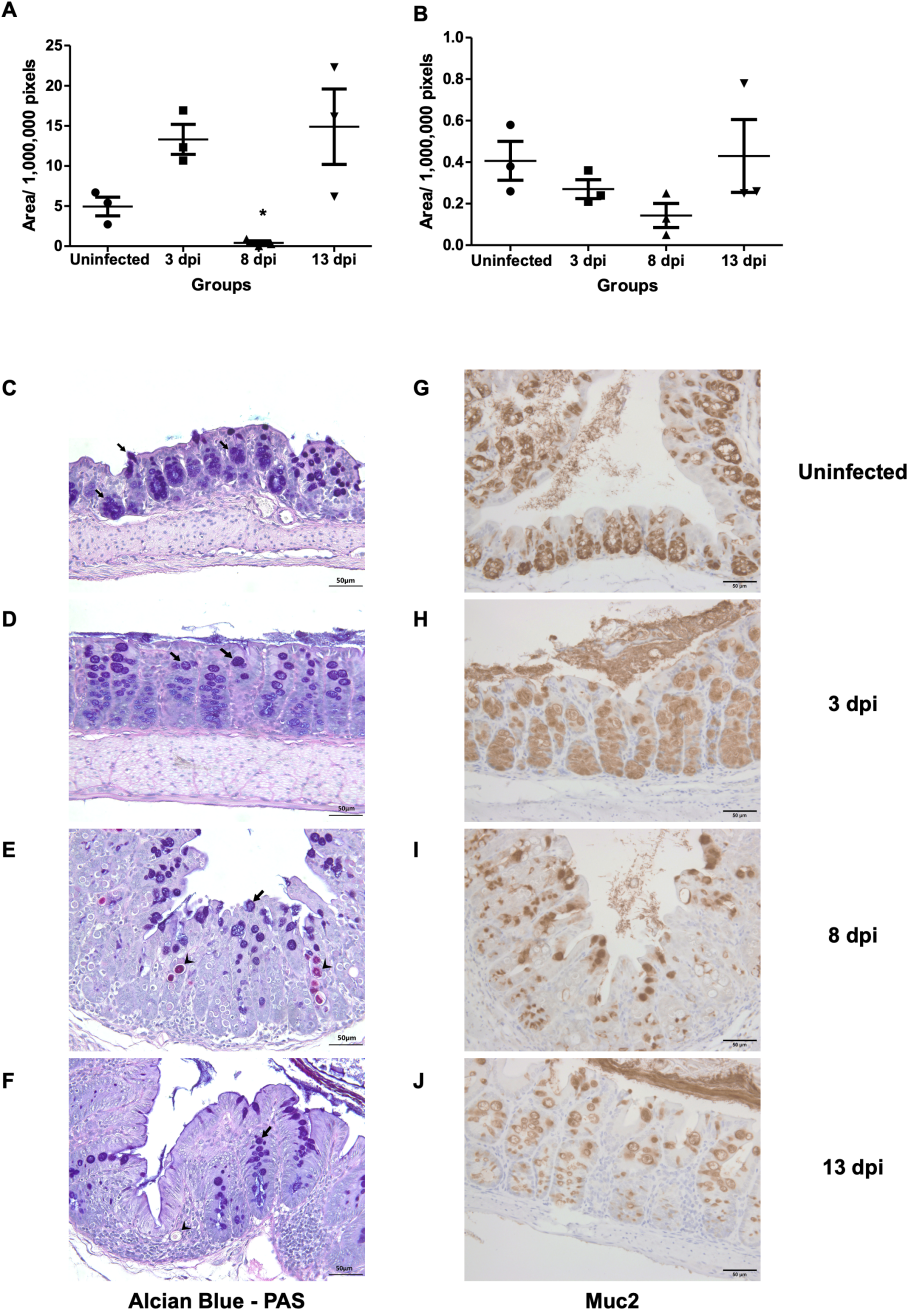
Goblet cell depletion and Muc2 expression following *E*. *pragensis* infection. **(A)** Quantification of goblet cell area based on PAS-Alcian Blue staining at 3, 8, and 13 dpi. **(B)** Quantification of Muc2-positive areas by immunohistochemistry (IHC) staining at 3, 8, and 13 dpi. **(C)** Representative PAS-Alcian Blue-stained sections of the large intestine from uninfected mice; **(D)** 3 dpi; **(E)** 8 dpi, and **(F)** 13 dpi mice. Black arrowheads indicate parasite (PAS positive). The black arrows indicate goblet cells (PAS positive and Alcian Blue positive). **(G)** Representative Muc2 IHC staining from uninfected mice; **(H)** 3 dpi; **(I)** 8 dpi; and **(J)** 13 dpi. Brown coloration (DAB staining) indicates Muc2 expression.

In Alcian Blue and PAS staining, goblet cell areas were still high at 3 dpi (13.31 ± 3.23 x 10^6^ pixels), as compared with uninfected control samples (4.94 ± 2.03 x10^6^ pixels). Then the goblet cells significantly decreased by 8 dpi (0.40 ± 0.45 x10^6^ pixels) and recovered at 13 dpi (14.89 ± 8.13x10^6^ pixels). Immunostaining of Muc2 also showed a reduction at 8 dpi (0.14 ± 0.10x10^6^ pixels), consistent with loss of mucosal barrier function at the peak of infection, before partial recovery at 13 dpi (**Figure 4**).

### 3.3 Host transcriptome analysis

**Figure 5:**
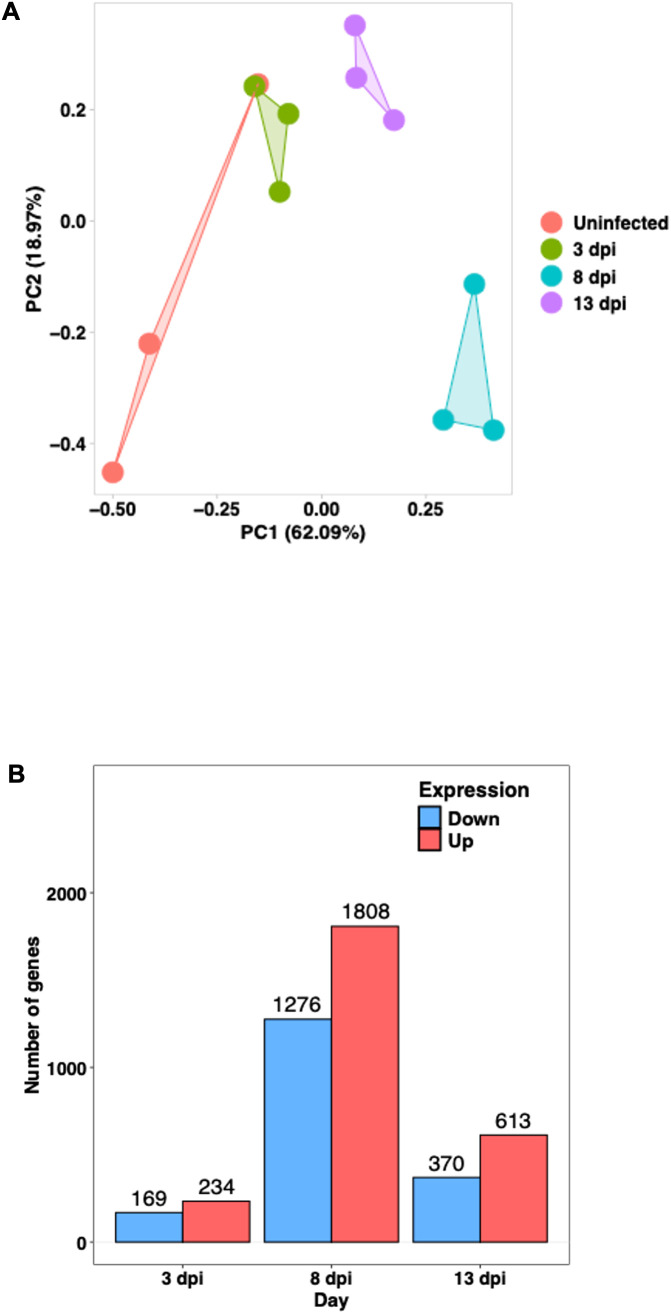
Transcriptomic profiles of the host large intestine during *E*. *pragensis* infection **(A)** Principal Component Analysis (PCA) of RNA-seq data. Each point represents an individual sample: red (uninfected), green (3 dpi), blue (8 dpi), and purple (13 dpi). **(B)** Number of differentially expressed genes (DEGs) at 3, 8, and 13 dpi, showing the number of upregulated (red) and downregulated (blue) genes.

Principal Component Analysis (PCA) showed that uninfected samples clustered tightly on the left side of the plot, whereas infected samples at 3, 8, and 13 dpi formed separate clusters along the PC1 axis, indicating a progressive shift in the transcriptomic profile over time. Notably, the 8 dpi group exhibited the greatest separation from the control group. The 13 dpi samples also formed a distinct cluster (**Figure 5A**).

**Figure 6:**
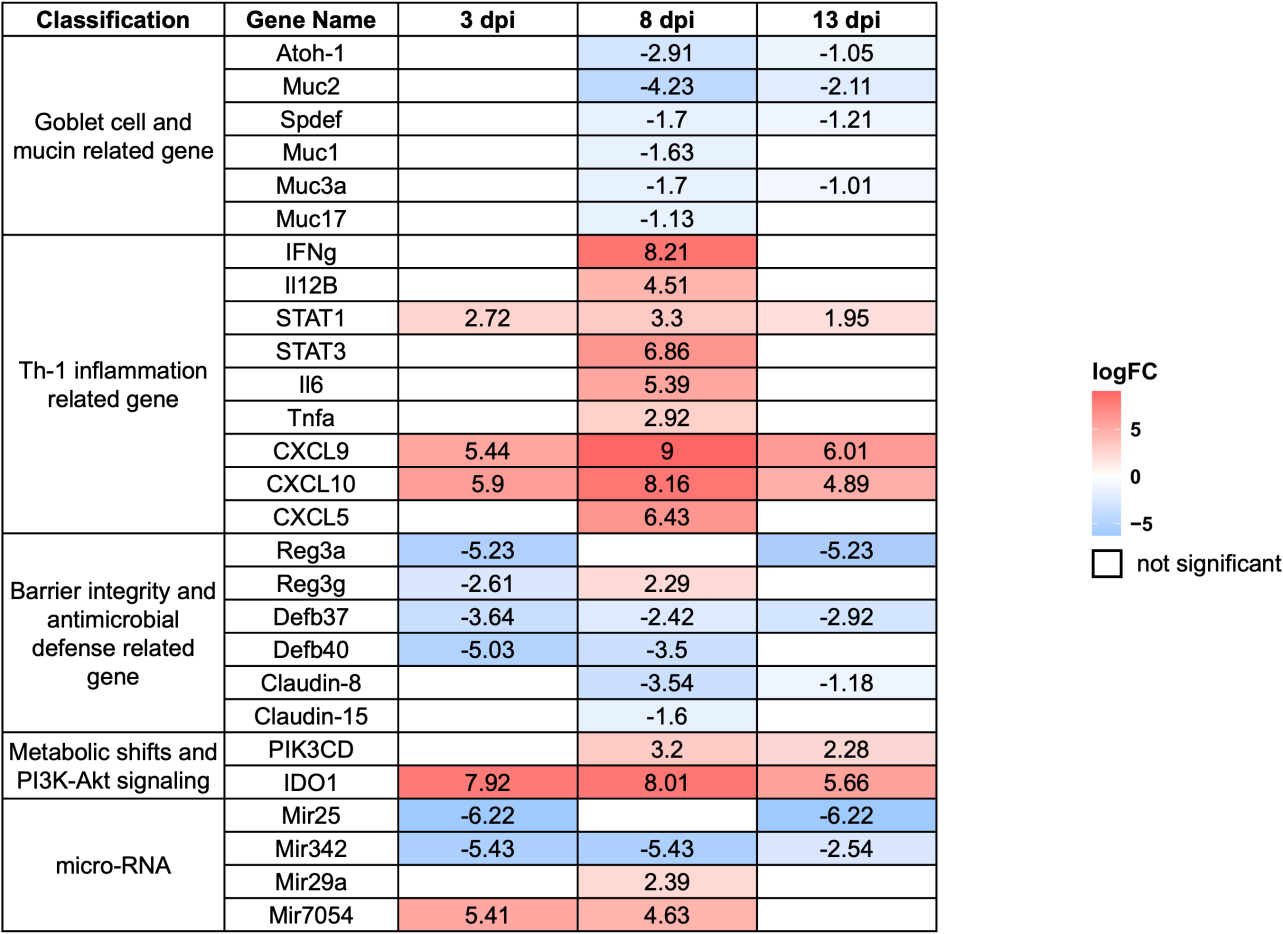
Expression profiles of selected differentially expressed genes (DEGs) in the host large intestine during *E. pragensis* infection. Heatmap displaying log_2_ fold-change values of selected DEGs at 3, 8, and 13 dpi relative to uninfected controls. Genes are grouped by functional categories: goblet cell and mucin-related genes, Th1-type inflammation-related genes, barrier integrity and antimicrobial defense-related genes, PI3K-Akt signaling-related genes, and microRNAs. Red indicates upregulation, and blue indicates downregulation. Genes outlined in white represent non-significant changes.

Based on fold-change calculations, a total of 403, 3,084, and 983 significantly differentially expressed genes (DEGs) were identified at 3, 8, and 13 dpi, respectively (**Figure 5B**). At 8 dpi, when the highest number of DEGs detected, there was strong upregulation of Interferon gamma (IFN-γ), Signal Transducer and Activator of Transcription 1 (STAT1), STAT3, Interleukin-6 (IL-6), and Tumor Necrosing Factor alpha (TNF-α), indicating a potent Th1-type inflammatory responses. Chemokines such as C-X-C motif chemokine ligand 9 (CXCL9), CXCL10, and CXCL5 were markedly upregulated, supporting continued immune cell infiltration. On the other hand, goblet cell-related genes including Muc2, Atonal homolog 1 (Atoh1), SAM pointed domain-containing ETS transcription factor (Spdef), and Muc3a were downregulated, highlighting substantial goblet cell dysfunction. Several antimicrobial genes and tight junction components such as Defensin beta 40 (Defb40), Claudin-8, and Claudin-15 were also reduced, indicating compromised mucosal barrier integrity. Notably, Indoleamine 2,3-dioxygenase 1 (IDO1) remained strongly upregulated, and microRNAs (miRNAs)Mir29a as well as Mir7054 were activated, suggesting further immune modulation (**Figure 6**).

At 3 dpi, immune activation was already evident. Expression of STAT1, CXCL9, and CXCL10 increased, indicating the initiation of IFN-γ signaling and recruitment of immune cells such as T cells and Natural Killer (NK) cells. IDO1 was highly upregulated, suggesting immune regulatory activity or early immune evasion mechanisms by the parasite. Simultaneously, the marked downregulation of Mir25 and Mir342 pointed to altered post-transcriptional regulation during this early phase. Genes associated with mucosal defense, including Regenerating islet-derived protein 3 alpha (Reg3a) and Defb40, were significantly suppressed, implying early epithelial barrier disruption and compromised antimicrobial activity (**Figure 6**).

By 13 dpi, gene expression patterns reflected a mixture of residual immune activity and delayed mucosal recovery. IFN-γ and STAT1 remained elevated, while chemokines such as CXCL9 and CXCL10 continued to be highly expressed, indicating sustained low-level inflammation. IDO1 remained upregulated, suggesting persistent local immune regulation. Interestingly, Claudin-15 expression increased, possibly reflecting efforts to restore epithelial barrier integrity. However, goblet cell markers such as Muc2, Spdef, and Muc3a remained downregulated, indicating persistent goblet cell dysfunction. Similarly, antimicrobial genes like Defb37, Defb40, and Reg3a continued to show reduced expression. The continued upregulation of Mir29a and Mir7054 may indicate roles in immune adaptation or epithelial remodeling during the resolution phase (**Figure 6**).

A heat map of selected DEGs was shown in **Figure 6** representing log2 fold-change values at 3 dpi, 8 dpi, and 13 dpi. The genes are grouped into functional categories, including goblet cell and mucin-related genes, Th1-type inflammation-related genes, barrier integrity and antimicrobial defense-related genes, PI3K-protein kinase B (Akt) signaling-related genes, and microRNAs (list of DEGs was shown in **Supplementary Table S2**).

### 3.4 *E. pragensis* transcriptome analysis

Parasite transcripts at 8 dpi were successfully mapped to the *E. falciformis* reference genome, with top 100 highly expressed genes identified. Genes were annotated using both ToxoDB and BLAST searches against the NCBI database. According to ToxoDB, 57 genes had defined annotations, though 43 genes were annotated as “unspecified products. In contrast, BLAST analysis using the NCBI database revealed that 47 genes had no significant homology, whereas 53 genes showed identifiable matches (**Supplementary Table S3**).

Genes potentially involved in the suppression of mucin production and intestinal mucosa integrity were subtilase domain-containing peptidases, known to degrade mucin and tight junctions, Notch and thrombospondin type 1 (TSP1) domain-containing protein, which may activate the Notch–Hes1 signaling pathway, and PAN/Apple domain-containing proteins, originally named after plasminogen, apple-like folding structures, and nematode proteins, known to interact with glycoproteins like mucin (**Table 1**). Although these transcripts were detected at 8 dpi, parasites in the earlier phase might express similar proteins.

**Table 1:** Candidate genes associated with mucin suppression.

Gene ID	Mean expression (Log Fold Change)	Domain
EfaB_MINUS_20412.g1838	14.91	Heat Shock Protein 90
EfaB_PLUS_22284.g1821,	14.61	PAN/Apple domain
EfaB_PLUS_800.g89	14.55	Peptidase S8/S53
EfaB_MINUS_34010.g2532,	14.40	PAN/Apple domain
EfaB_PLUS_9917.g893	14.08	Thioredoxin/PDI
EfaB_PLUS_23143.g1929	13.76	PAN/Apple domain
EfaB_MINUS_49504.g2799	13.75	GAPDH
EfaB_PLUS_13227.g1152	12.52	Cyclophilin
EfaB_MINUS_20371.g1783	12.19	Peptidase S8/S53
EfaB_PLUS_1425.g183	11.96	Notch + TSP1 domain

Here we show that host genes associated with T helper 1 (Th1) type inflammation, epithelial barrier disruption, and immune regulation were up-regulated as early as 3 dpi, when the amount of *E. pragensis* in the intestinal mucosa was minimal. *E. pragensis* in the mucosa produced enzymes that were known to degrade mucin and tight junctions of the epithelial layer and produced proteins that could activate the Notch signaling pathway and Hairy and Enhancer of Split-1(Notch–Hes1) signaling pathway.

### 3.5 Th-1 related gene expression

To validate the transcriptomic findings, we performed quantitative PCR targeting key immune genes includes STAT1, Tbx21, and Il12b (**Supplementary Figure 1**). All genes showed peak expression at 8 dpi. Expression levels declined by 13 dpi, consistent with RNA-seq DEGs data (**Figure 6**; **Supplementary Table 2**).

## 4 Discussion

The present study highlights the dynamics of *E. pragensis* infection in mice, regarding parasite proliferation, goblet cell function, mucin regulation, and the subsequent colitis caused by the parasite. Oocyst excretion peaked at 8–9 dpi and ended at 13 dpi. Histopathological analysis revealed an elevated histopathological score and a significant reduction in goblet cell numbers at 8 dpi, coinciding with the peak of parasite proliferation in the proximal-median colon.

The invasion of *E. pragensis* into the colonic epithelium likely contributes to colitis, as the disruption of goblet cell function and mucin production compromises the intestinal barrier. The decrease in goblet cells during peak infection suggests that the parasite breaches the mucosal barrier, facilitating its deeper invasion and leading to the inflammation and tissue damage characteristic of colitis. This breakdown of the mucosal barrier, which has been observed in other gastrointestinal infections (Pelaseyed et al., 2014), likely exacerbates the inflammation and worsens colitis severity. It is widely known that goblet cells play an essential role in maintaining epithelial integrity by secreting mucins, which form a protective layer against pathogens (Dkhil et al., 2013; Grondin et al., 2020; Uemura et al., 2022).

To gain a deeper understanding of the molecular mechanisms involved in *E. pragensis* infection in mice, we examined RNA transcripts of both host and parasite throughout the infection. RNA sequencing (RNA-seq) technology enables us to identify differentially expressed genes and non-coding elements, associated with epithelial barrier function, immune modulation, and goblet cell differentiation. It should offer new insights into host-parasite interactions and mucosal pathogenesis (Ehret et al., 2017; Gallego-Lopez et al., 2022; Kim et al., 2022).

In this study, RNA-seq analysis revealed dynamic changes in host gene expression throughout the course of *E. pragensis* infection. We found that, at 3 dpi when mucosal parasite number was minimal, genes for mucosal barrier function-related proteins were already downregulated, and those for Th1-related proteins were upregulated. These facts indicate that Th1 type inflammation was already elicited, and the barrier function of large intestine was disrupted in this early phase, though no histological changes were detected.

At 8 dpi, when the inflammation peaked, a marked downregulation of key goblet cell-associated genes such as Atoh-1, Spdef, and several mucins (Muc2, Muc1, Muc17, Muc3a) was observed, supporting histological findings of goblet cell depletion and disruption of the mucosal barrier. Concomitantly, genes involved in inflammatory and interferon-related responses, including IFN-γ, IL-12b, STAT1, STAT3, IL-6, TNF-α, CXCL9, CXCL10, and CXCL5, were significantly upregulated, indicating a strong pro-inflammatory environment during peak infection. These molecules are known to drive immune cell recruitment and activation, contributing to inflammation and tissue damage in protozoan infections (Clark et al., 2017; Leon-Coria et al., 2018; Uemura et al., 2022). The increased expression of Phosphoinositide-3-Kinase Catalytic Subunit Delta (PIK3CD), a catalytic subunit of phosphoinositide 3-kinase delta (PI3K-δ) signaling, further supports the activation of immune and epithelial signaling pathways involved in inflammation and epithelial stress responses (Okkenhaug and Vanhaesebroeck, 2003; Jalil et al., 2023).

At the peak of the infection, genes related to antimicrobial defense and barrier function, including Reg3a, Reg3g, Defb37, Defb40, Claudin-8, and Claudin-15, also showed altered expression, suggesting weakened antimicrobial protection and impaired tight junction integrity. Similar disruption of epithelial barrier genes has been reported in other enteric protozoal infections and is often associated with increased permeability and dysbiosis (Camilleri et al., 2012; Pelaseyed et al., 2014; Zhou et al., 2022).

By 13 dpi, both Atoh1 and Muc2 expression levels recovered, suggesting a host-driven compensatory mechanism to restore goblet cell function and rebuild the mucosal barrier. This pattern resembles responses seen in other parasitic infections where epithelial regeneration follows peak inflammation (Dkhil et al., 2013; Hechenbleikner and McQuade, 2015; Figueiredo-Campos et al., 2018). The inverse relationship between parasite density and goblet cell activity supports this interpretation, highlighting the dynamic balance between host defense and parasite persistence (Gustafsson and Johansson, 2022; Uemura et al., 2022).

This subclinical mucosal damage, not detectable by histology but suggested by gene expression changes, likely contributed to the massive inflammation observed at 8 dpi, was supposedly caused by the proteins produced by *E. pragensis* in the intestinal mucosa. We found that *E*. *pragensis* expressed two genes coding subtilisin-like serine proteases (Peptidase S8/S53 domain). Although direct evidence for mucin degradation by Eimeria is lacking, studies in other parasites have shown that serine proteases secreted by the nematode *Trichuris muris* degrade the intestinal mucin Muc2, leading to a more porous mucus barrier that facilitates parasite survival and persistence (Hasnain et al., 2012).

Three genes (EfaB_PLUS_22284.g1821, EfaB_MINUS_34010.g2532, EfaB_PLUS_23143.g1929) encode proteins containing PAN/Apple domains, which are recognized for mediating interactions with glycoproteins such as mucins. The P104 protein in *Toxoplasma gondii* contains a PAN/Apple domain, that has been shown to bind to sulfated proteoglycans such as chondroitin sulfate A (CSA) and chondroitin sulfate C (CSC) on the surface of host cells. This interaction is important for parasite adhesion and invasion (Gong et al., 2012).

In particular, one of the detected genes was for Notch and TSP1 domains. Activation of the Notch–Hes1 pathway suppresses Atoh1, a key transcription factor for goblet cell differentiation, thereby downregulating Muc2 expression. The presence of such a domain suggests a possible parasite-mediated mimicry or interference with host differentiation pathways (Fre et al., 2005).

Although parasite transcriptomic data were collected only at 8 dpi in this study, based on the findings of Ehret et al. (2017), which showed a stable parasite transcriptome regardless of host immune status, we speculate that gene expression likely remained consistent at 3 and 13 dpi as well.

These results suggested that the initial parasite-host interactions occurring at 3 dpi triggered a mucosal barrier dysfunction, leading to the Th1-type inflammatory responses. Our model provides valuable insight into the mechanisms of mucosal barrier disruption during protozoan infection, and possibly in inflammatory bowel disease (IBD) as well (Pelaseyed et al., 2014; Liu et al., 2016; Sun et al., 2024).

The qPCR results for STAT1, Tbx21, and Il12b were consistent with RNA-seq data, confirming the upregulation of Th1-related genes during the peak of *E. pragensis* infection. The similar expression trends observed in both methods validate the reliability of the transcriptomic analysis and strengthen the conclusion that Th1 immune activation is involved in the host response at 8 dpi.

Taken together, these transcriptomic findings provide a comprehensive molecular framework for understanding the multifactorial impact of *E*. *pragensis* infection. Our findings demonstrate that *E. pragensis* causes colitis by disrupting goblet cell function, impairing mucin production, and triggering inflammation marked by Th1-related gene expression. The combined effects of epithelial weakening and immune activation promote parasite persistence and tissue damage.

While our data suggest that goblet cell depletion and mucin gene downregulation are directly associated with *E. pragensis* proliferation and inflammation, the potential contribution of intestinal microbiota cannot be excluded. In *Giardia muris*-infected mice, protozoan-induced dysbiosis has been shown to impair mucin glycosylation by downregulating host glycosyltransferases (Fekete et al., 2024). Similarly, infections with *E. acervulina* and *E. maxima* have been reported to alter gut microbial diversity, reduce short-chain fatty acid–producing bacteria, and increase opportunistic pathogens, changes that coincide with peak parasite burden and epithelial damage (Campos et al., 2024; Liu et al., 2024). These findings highlight a possible synergistic role of parasite activity and microbial imbalance in disrupting the mucosal barrier that potentially leading to systemic endotoxemia. Although this mechanism was not assessed in our study, it is highly relevant to mucosal pathology (Di Vincenzo et al., 2024). Moreover, while we did not perform microbiota profiling or evaluate bacterial translocation, it is plausible that *E. pragensis* similarly induces dysbiosis, exacerbating goblet cell depletion and mucin layer erosion.

In addition to microbiota factors, other biological variables may also influence host responses. In this study, we used male mice, based on established protocols in previous *E. pragensis* infection studies. However, sex-based differences were not evaluated, despite evidence that female mice may exhibit stronger Th1 responses (Alonaizan et al., 2021). In fact, male mice have been shown to be more susceptible to *Eimeria* infection than female mice, which was associated with lower number of goblet cells and less expression of MUC2 mRNA in male mice (Dkhil, 2015).

These limitations should be addressed in future studies to provide a more comprehensive understanding of host responses to *E. pragensis* infection. Further investigation of key genes, regulatory molecules, and the role of microbiota will enhance our understanding of the multifactorial mechanisms driving mucosal disruption during protozoan infection. These insights may contribute to the development of new strategies for managing parasitic colitis and mucosal disease.

## Data Availability

The original contributions presented in the study are publicly available. This data can be found here: NCBI BioProject accession PRJDB20716.

## Supplementary material

Supplementary Figure 1 Th-1 gene expression during *E. pragensis* infection. qPCR fold changes of **(A)** STAT1, **(B)** Il12b, and **(C)** Tbx21 at 3, 8, and 13 dpi. Data are shown as fold change relative to uninfected controls. Error bars indicate SEM.

## References

[B1] Alonaizan R. Woods S. Hargrave K. E. Roberts C. W. (2021). An exaggerated immune response in female Balb/c mice controls initial *Toxoplasma gondii* multiplication but increases mortality and morbidity relative to male mice. Pathogens 10, 1–12. doi: 10.3390/pathogens10091154 PMC8470933 34578186

[B2] Bhattacharyya S. Ratajczak C. K. Vogt S. K. Kelley C. Colonna M. Schreiber R. D.. (2010). TAK1 targeting by glucocorticoids determines JNK and IκB regulation in toll-like receptor-stimulated macrophages. Blood 115, 1921–1931. doi: 10.1182/blood-2009-06-224782 20065289 PMC2837326

[B3] Blake D. P. Tomley F. M. (2014). Securing poultry production from the ever-present Eimeria challenge. Trends in Parasitology 30 (1), 12–19. doi: 10.1016/j.pt.2013.10.003 24238797

[B4] Blake D. P. Hesketh P. Archer A. Shirley M. W. Smith A. L (2006). *Eimeria maxima*: The influence of host genotype on parasite reproduction as revealed by quantitative real-time PCR. International Journal for Parasitology 36 (1), 97–105. doi: 10.1016/j.ijpara.2005.09.011 16300767

[B5] Bolger A. M. Lohse M. Usadel B. (2014). Trimmomatic: A flexible trimmer for Illumina sequence data. Bioinformatics 30, 2114–2120. doi: 10.1093/bioinformatics/btu170 24695404 PMC4103590

[B6] Borisova M. A. Achasova K. M. Morozova K. N. Andreyeva E. N. Litvinova E. A. Ogienko A. A.. (2020). Mucin-2 knockout is a model of intercellular junction defects, mitochondrial damage and ATP depletion in the intestinal epithelium. Sci. Rep. 10, 1–17. doi: 10.1038/s41598-020-78141-4 PMC7713437 33273633

[B7] Burrell A. Tomley F. M. Vaughan S. Marugan-Hernandez V. (2020). Life cycle stages, specific organelles and invasion mechanisms of Eimeria species. Parasitology 147, 263–278. doi: 10.1017/S0031182019001562 31727204 PMC10317661

[B8] Camilleri M. Madsen K. Spiller R. Van Meerveld B. Verne G. (2012). Intestinal barrier function in health and gastrointestinal disease. Neurogastroenterol. Motility. 24, 503–512. doi: 10.1111/j.1365-2982.2012.01921.x PMC5595063 22583600

[B9] Campos P. M. Miska K. B. Jenkins M. C. Yan X. Proszkowiec-Weglarz M. (2024). Effects of Eimeria acervulina infection on the luminal and mucosal microbiota of the cecum and ileum in broiler chickens. Sci. Rep. 14, 1–13. doi: 10.1038/s41598-024-61299-6 PMC11087572 38729976

[B10] Chapman H. D. (2014). Milestones in avian coccidiosis research: A review. Poultry Science. 93, 501–511. doi: 10.3382/ps.2013-03634 24604841

[B11] Clark E. L. Tomley F. M. Blake D. P. (2017). Are eimeria genetically diverse, and does it matter? Trends Parasitology. 33, 231–241. doi: 10.1016/j.pt.2016.08.007 27593338

[B12] Di Vincenzo F. Del Gaudio A. Petito V. Lopetuso L. R. Scaldaferri F. (2024). Gut microbiota, intestinal permeability, and systemic inflammation: a narrative review. Internal Emergency Med. 19, 275–293. doi: 10.1007/s11739-023-03374-w PMC10954893 37505311

[B13] Dkhil M. A. (2015). Sex-determined susceptibility and differential MUC2 mRNA expression during the course of murine intestinal eimeriosis. Parasitol. Res. 114, 283–288. doi: 10.1007/s00436-014-4192-2 25349142

[B14] Dkhil M. A. Delic D. Al-Quraishy S. (2013). Goblet cells and mucin related gene expression in mice infected with Eimeria papillate. Sci. World J. 2003, 1–6. doi: 10.1155/2013/439865 PMC3866723 24367242

[B15] Dobin A. Davis C. A. Schlesinger F. Drenkow J. Zaleski C. Jha S.. (2013). STAR: Ultrafast universal RNA-seq aligner. Bioinformatics 29, 15–21. doi: 10.1093/bioinformatics/bts635 23104886 PMC3530905

[B16] Ehret T. Spork S. Dieterich C. Lucius R. Heitlinger E. (2017). Dual RNA-seq reveals no plastic transcriptional response of the coccidian parasite Eimeria falciformis to host immune defenses. BMC Genomics 18, 1–17. doi: 10.1186/s12864-017-4095-6 PMC5584376 28870168

[B17] Ewels P. Magnusson M. Lundin S. Käller M. (2016). MultiQC: Summarize analysis results for multiple tools and samples in a single report. Bioinformatics 32, 3047–3048. doi: 10.1093/bioinformatics/btw354 27312411 PMC5039924

[B18] Fekete E. Allain T. Sosnowski O. Anderson S. Lewis I. A. Buret A. G. (2024). Giardia spp.-induced microbiota dysbiosis disrupts intestinal mucin glycosylation. Gut Microbes 16, 2412676. doi: 10.1080/19490976.2024.2412676 39412866 PMC11485787

[B19] Figueiredo-Campos P. Ferreira C. Blankenhaus B. Veldhoen M. (2018). Eimeria vermiformis infection model of murine small intestine. Bio-Protocol. 8, 1–10. doi: 10.21769/bioprotoc.3122 PMC6485400 31032380

[B20] Fre S. Huyghe M. Mourikis P. Robine S. Louvard D. Aetavanis-Tsakonas S. (2005). Notch signals control the fate of immature progenitor cells in the intestine. Nature 435, 964–968. doi: 10.1038/nature03589 15959516

[B21] Gallego-Lopez G. M. Cavazos C. Tibabuzo P. A. Garfoot A. O’Connor R. Knoll L. (2022). Dual transcriptomics to determine gamma interferon- independent host response to intestinal cryptosporidium parvum infection. Infection Immunity. 90, 1–18. doi: 10.1128/iai.00638-21 PMC8852703 34928716

[B22] Ghasemi A. Zahediasl S. (2012). Normality tests for statistical analysis: A guide for non-statisticians. Int. J. Endocrinol. Metab. 10, 486–489. doi: 10.5812/ijem.3505 23843808 PMC3693611

[B23] Gong H. Kobayashi K. Sugi T. Takemae H. Kurokawa H. Horimoto T.. (2012). A novel PAN/apple domain-containing protein from Toxoplasma gondii: Characterization and receptor identification. PloS One 7, 1–10. doi: 10.1371/journal.pone.0030169 PMC3261864 22276154

[B24] Grondin J. A. Kwon Y. Far P. Haq S. Khan W. (2020). Mucins in intestinal mucosal defense and inflammation: learning from clinical and experimental studies. Front. Immunol. 11. doi: 10.3389/fimmu.2020.02054 PMC7500085 33013869

[B25] Günay-Esiyok Ö. Gupta N. (2024). “Eimeria falciformis,” in Trends in Parasitology (London, UK: Elsevier Ltd). doi: 10.1016/j.pt.2024.09.008 PMC7616687 39362799

[B26] Gustafsson J. K. Johansson M. E. V. (2022). The role of goblet cells and mucus in intestinal homeostasis. Nat. Rev. Gastroenterol. Hepatology. 19, 785–803. doi: 10.1038/s41575-022-00675-x 36097076

[B27] Hasnain S. Z. McGuckin M. Grencis R. Thornton D. (2012). Serine protease(s) secreted by the nematode Trichuris muris degrade the mucus barrier. PloS Negl. Trop. Diseases. 6, 1–13. doi: 10.1371/journal.pntd.0001856 PMC3469553 23071854

[B28] Hechenbleikner E. M. McQuade J. A. (2015). Parasitic colitis. Clinics Colon Rectal Surg. 28, 79–86. doi: 10.1055/s-0035-1547335 PMC4442724 26034403

[B29] Jalil A. T. Hassan N. Abdulameer S. Farhan Z. Sulaeman A. Al-Azzawi A.. (2023). Phosphatidylinositol 3-kinase signaling pathway and inflammatory bowel disease: Current status and future prospects. Fundam. Clin. Pharmacol. 37, 910–917. doi: 10.1111/fcp.12894 36939850

[B30] Kim M. Chung Y. Manjula P. Seo D. Cho S. Cho E.. (2022). Time-series transcriptome analysis identified differentially expressed genes in broiler chicken infected with mixed Eimeria species. Front. Genet. 13. doi: 10.3389/fgene.2022.886781 PMC9393255 36003329

[B31] Kim Y. S. Ho S. B. (2010). Intestinal goblet cells and mucins in health and disease: Recent insights and progress. Curr. Gastroenterol. Rep. 12, 319–330. doi: 10.1007/s11894-010-0131-2 20703838 PMC2933006

[B32] Kim D. Paggi J. Park C. Bennet C. Salzberg S. (2019). Graph-based genome alignment and genotyping with HISAT2 and HISAT-genotype. Nat. Biotechnol. 37, 907–915. doi: 10.1038/s41587-019-0201-4 31375807 PMC7605509

[B33] Kubista M. Andrade J. M. Bengtsson M. Forootan A. Jonák J. Lind K (2006). The real-time polymerase chain reaction. Molecular Aspects of Medicine 27, 95–125. doi: 10.1016/j.mam.2005.12.007 16460794

[B34] Kyunai Y. M. Sakamoto M. Koreishi M. Tsujino Y. Satoh A. (2023). Fucosyltransferase 8 (FUT8) and core fucose expression in oxidative stress response. PloS One 18, 1–12. doi: 10.1371/journal.pone.0281516 PMC9924996 36780470

[B35] Leon-Coria A. Kumar M. Chadee K. (2020). The delicate balance between Entamoeba histolytica, mucus and microbiota. Gut Microbes 11, 118–125. doi: 10.1080/19490976.2019.1614363 31091163 PMC6973333

[B36] Leon-Coria A. Kumar M. Moreau F. Chadee K. (2018). Defining cooperative roles for colonic microbiota and Muc2 mucin in mediating innate host defense against Entamoeba histolytica. PloS Pathogens. 14, 1–22. doi: 10.1371/journal.ppat.1007466 PMC6268003 30500860

[B37] Li Y. Su Z. Zhao W. Zhang X. Momin N. Zhang C.. (2020). Multifunctional oncolytic nanoparticles deliver self-replicating IL-12 RNA to eliminate established tumors and prime systemic immunity. Nat. cancer. 1, 882–893. doi: 10.1038/s43018-020-0095-6 34447945 PMC8386348

[B38] Liao Y. Smyth G. K. Shi W. (2014). FeatureCounts: An efficient general purpose program for assigning sequence reads to genomic features. Bioinformatics 30, 923–930. doi: 10.1093/bioinformatics/btt656 24227677

[B39] Liu J. Guo J. Whitmore M. A. Tobin I. Kim D. M. Zhao Z.. (2024). Dynamic response of the intestinal microbiome to Eimeria maxima- induced coccidiosis in chickens. Microbiol. Spectrum. 12, 1–16 doi: 10.1128/spectrum.00823-24 PMC11448223 39248475

[B40] Liu S. Wang L. Zheng H. Xu Z. Roellig D. Li N.. (2016). Comparative genomics reveals Cyclospora cayetanensis possesses coccidia-like metabolism and invasion components but unique surface antigens. BMC Genomics 17, 1–17. doi: 10.1186/s12864-016-2632-3 27129308 PMC4851813

[B41] Lu C. Yan Y. Jian F. Ning C. (2021). Coccidia-microbiota interactions and their effects on the host. Front. Cell. Infection Microbiol. 11. doi: 10.3389/fcimb.2021.751481 PMC8517481 34660347

[B42] Okkenhaug K. Vanhaesebroeck B. (2003). PI3K in lymphocyte development, differentiation and activation. Nat. Rev. Immunol. 3, 317–330. doi: 10.1038/nri1056 12669022

[B43] Pelaseyed T. Bergström J. H. Gustafsson J. K. Ermund A. Birchenough G. M. H. Schütte A.. (2014). The mucus and mucins of the goblet cells and enterocytes provide the first defense line of the gastrointestinal tract and interact with the immune system. Immunol. Rev. 260, 8–20. doi: 10.1111/imr.12182 24942678 PMC4281373

[B44] Qing F. Tian H. Wang B. Xie B. Sui L. Xie X.. (2024). Interferon regulatory factor 7 alleviates the experimental colitis through enhancing IL-28A-mediated intestinal epithelial integrity. J. Trans. Med. 22, 905. doi: 10.1186/s12967-024-05673-y PMC11457333 39370517

[B45] Robinson M. D. McCarthy D. J. Smyth G. K. (2009). edgeR: A Bioconductor package for differential expression analysis of digital gene expression data. Bioinformatics. 26 (1), 139–140. doi: 10.1093/bioinformatics/btp616 PMC2796818 19910308

[B46] Schneider C. A. Rasband W. S. Eliceiri K. W. (2012). NIH Image to ImageJ: 25 years of image analysis. Nat. Methods 9, 671–675. doi: 10.1038/nmeth.2089 22930834 PMC5554542

[B47] Siswandi R. Yoshida A. Satoh H. Nonaka N. (2018). “Eimeria pragensis induce immune mediated intestinal hypomotility in C57BL/6 mice,” in *Proceedings of the 20th FAVA Congress & The 15th KIVNAS PDHI, Bali Nov 1–3, 2018* , Denpasar, Bali, Indonesia: Federation of Asian Veterinary Associations (FAVA) and Perhimpunan Dokter Hewan Indonesia (PDHI).

[B48] Siswandi R. Yoshida A. Satoh H. Nonaka N. (2019). X-ray evaluation of intestinal dysmotility induced by Eimeria pragensis infection in C57BL/6 mice. J. Veterinary Med. Sci. 81, 1021–1028. doi: 10.1292/jvms.19-0137 PMC6656811 31118353

[B49] Sun Y. Liu P. Guo W. Guo J. Chen J. Xue X.. (2024). Study on the alleviative effect of Lactobacillus plantarum on Eimeria falciformis infection. Infection Immunity. 92, 1–17. doi: 10.1128/iai.00130-24 PMC11324035 38842306

[B50] Tang D. Liu S. Sun H. Qin X. Zhou N. Zheng W.. (2020). All-trans-retinoic acid shifts Th1 towards Th2 cell differentiation by targeting NFAT1 signalling to ameliorate immune-mediated aplastic anaemia. Br. J. Haematology. 191, 906–919. doi: 10.1111/bjh.16871 32729137

[B51] Uemura R. Kawakado M. Sueyoshi M. Nonaka N. Horii Y. (2022). Eimeria pragensis infection alters the gut microenvironment to favor extrinsic shiga toxin-producing Escherichia coli O157:H7 colonization in mice. Parasitol. Int. 87, 1–6. doi: 10.1016/j.parint.2021.102521 34856387

[B52] Westermann A. J. Barquist L. Vogel J. (2017). Resolving host–pathogen interactions by dual RNA-seq. PloS Pathog. 13, 1–19. doi: 10.1371/journal.ppat.1006033 PMC5313147 28207848

[B53] Yunus M. Horii Y. Makimura S. Smith A. (2005a). Murine goblet cell hypoplasia during Eimeria pragensis infection is ameliorated by clindamycin treatment. J. Veterinary Med. Sci. 67, 311–315. doi: 10.1292/jvms.67.311 15805736

[B54] Yunus M. Horii Y. Makimura S. Smith A. (2005b). The relationship between the anticoccidial effects of clindamycin and the development of immunity in the Eimeria pragensis/mouse model of large intestinal coccidiosis. J. Veterinary Med. Sci. 67, 165–170. doi: 10.1292/jvms.67.165 15750312

[B55] Zhou A. Yuan Y. Yang M. Huang Y. Li X. Li S.. (2022). Crosstalk between the gut microbiota and epithelial cells under physiological and infectious conditions. Front. Cell. Infection Microbiol. 12. doi: 10.3389/fcimb.2022.832672 PMC8829037 35155283

