# Supplementary figures and images for: Down-regulation of colon mucin production induced by Eimeria pragensis infection in mice
Source: Front Parasitol. 2025 Jun 24;4:1621486. doi: 10.3389/fpara.2025.1621486 (PMC12234464; doi:10.3389/fpara.2025.1621486)

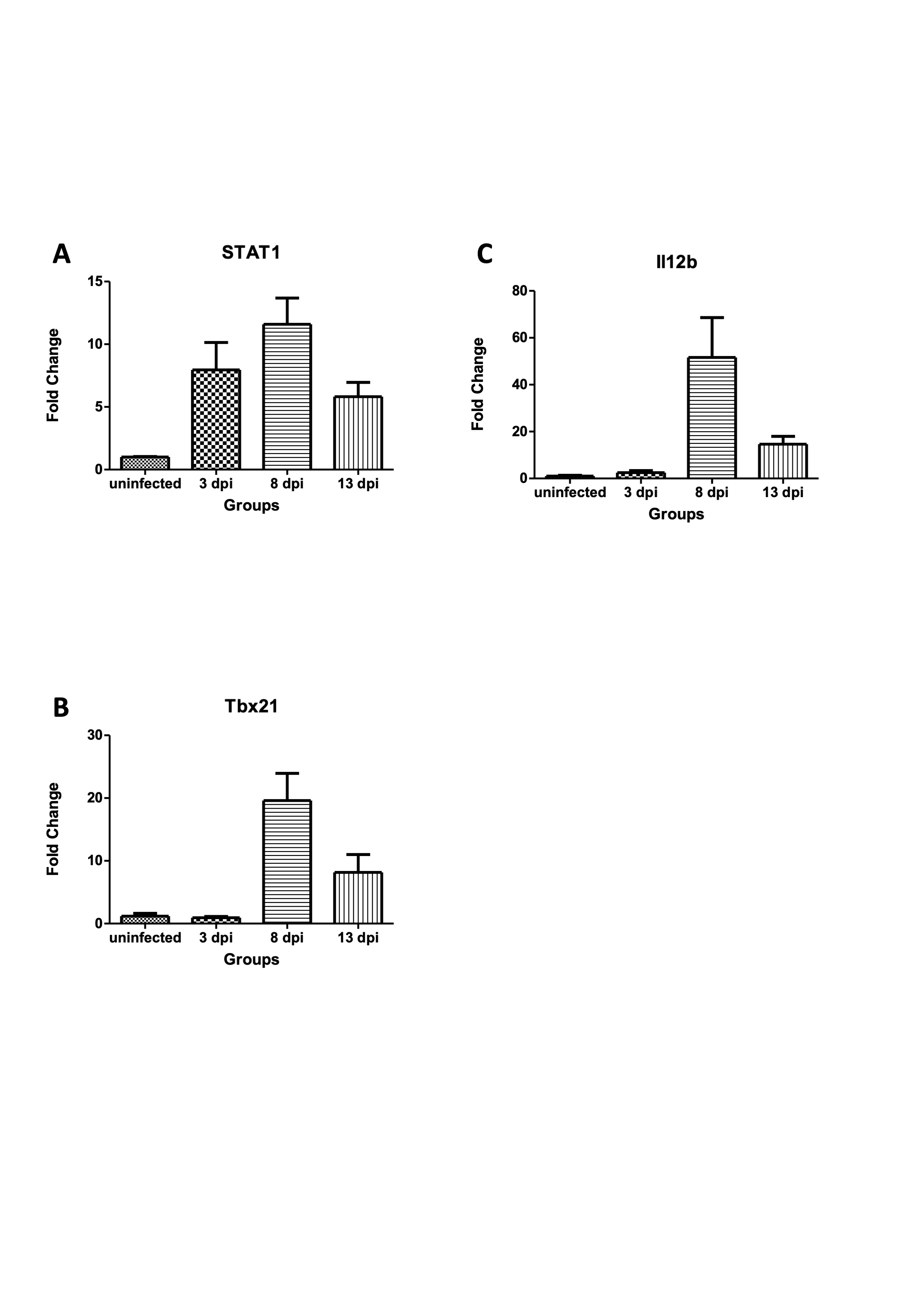

Supplement: Supplementary Figure 1 — Th-1 gene expression during E. pragensis infection. qPCR fold changes of (A) STAT1, (B) Il12b, and (C) Tbx21 at 3, 8, and 13 dpi. Data are shown as fold change relative to uninfected controls. Error bars indicate SEM. [file Image1.tiff]
