# Supplementary material for: Down-regulation of colon mucin production induced by Eimeria pragensis infection in mice
Source: Front Parasitol. 2025 Jun 24;4:1621486. doi: 10.3389/fpara.2025.1621486 (PMC12234464; doi:10.3389/fpara.2025.1621486)
Supplement: Supplementary file 2 [file SupplementaryFile1.pdf]

Supplementary Material

1 Supplementary Tables

Supplementary Table 1. Mapping Ratio of *E. pragensis* RNA-sequencing

Sample ID	Uniquely mapped reads number	Uniquely mapped reads %
13dpi_1	1397	0.01%
13dpi_2	1629	0.01%
13dpi_3	2244	0.01%
3dpi_1	4307	0.03%
3dpi_2	337	0.00%
3dpi_3	3208	0.02%
8dpi_1	3966146	24.56%
8dpi_2	3738404	24.36%
8dpi_3	981166	7.25%

Supplementary Table 2. Differential Expression Genes (DEGs) of Host RNA-seq

See the uploaded Excel file.

- Tab 1: 3 dpi
- Tab 2: 8 dpi
- Tab 3: 13 dpi

Supplementary Table 3. Top 100 highly expressed gene *E. pragensis*

No	GeneID	Mean Expression	ToxoDB result	NCBI Blast result
1	**EfaB_PLUS_43143.g2625**	17.37	unspecified product	*Eimeria acervulina* GPI transamidase subunit PIG-U, putative partial mRNA
2	**EfaB_PLUS_4114.g413**	16.63	unspecified product	No significant similarity found.
3	**EfaB_PLUS_28918.g2252**	16.32	unspecified product	*Eimeria nieschulzi* gametocyte antigen 2 (gam56-2) and gametocyte antigen 1 (gam56-1) genes, complete cds, alternatively spliced; and gametocyte protein (gam82) gene, complete cds
4	**EfaB_MINUS_10843.g935**	16.19	unspecified product	No significant similarity found.
5	**EfaB_PLUS_28918.g2251**	15.63	unspecified product	*Eimeria nieschulzi* gametocyte antigen 2 (gam56-2) and gametocyte antigen 1 (gam56-1) genes, complete cds, alternatively spliced; and gametocyte protein (gam82) gene, complete cds
6	**EfaB_PLUS_13441.g1177**	15.54	unspecified product	No significant similarity found.
7	**EfaB_MINUS_6929.g629**	14.98	Alanine dehydrogenase/pyridine nucleotide transhydrogenase NAD(H)-binding domain, Alanine dehydrogenase/pyridine nucleotide transhydrogenase N-terminal, Methyltransferase domain-containing protein	No significant similarity found.
8	**EfaB_MINUS_20412.g1838**	14.91	Heat shock protein Hsp90 family, Histidine kinase/HSP90-like ATPase domain-containing protein	*Eimeria maxima* heat shock protein 90, putative partial mRNA
9	**EfaB_PLUS_22284.g1821**	14.61	PAN/Apple domain-containing protein	No significant similarity found.

10	**EfaB_PLUS_3469.g360**	14.58	unspecified product	No significant similarity found.
11	**EfaB_PLUS_800.g89**	14.55	Peptidase S8/S53 domain-containing protein	MAG: *Planctomycetota bacterium* isolate bin43 chromosome
12	**EfaB_PLUS_28918.g2250**	14.52	unspecified product	*Eimeria nieschulzi* gametocyte antigen 2 (gam56-2) and gametocyte antigen 1 (gam56-1) genes, complete cds, alternatively spliced; and gametocyte protein (gam82) gene, complete cds
13	**EfaB_MINUS_7048.g638**	14.45	unspecified product	No significant similarity found.
14	**EfaB_MINUS_34010.g2532**	14.40	PAN/Apple domain-containing protein	No significant similarity found.
15	**EfaB_MINUS_22450.g1954**	14.11	unspecified product	*Eimeria intestinalis* elongation factor 1-alpha mRNA, partial cds
16	**EfaB_PLUS_9917.g893**	14.08	Thioredoxin domain-containing protein	*Toxoplasma gondii* mRNA for putative protein disulfide isomerase (pdi gene)
17	**EfaB_PLUS_32658.g2380**	14.02	Tubulin/FtsZ GTPase domain, Tubulin/FtsZ 2-layer sandwich domain-containing protein	*Eimeria tenella* beta-tubulin mRNA, complete cds
18	**EfaB_PLUS_2074.g242**	13.92	Sporulated oocyst TA4 antigen domain-containing protein	No significant similarity found.
19	**EfaB_MINUS_14978.g1258**	13.86	unspecified product	No significant similarity found.
20	**EfaB_PLUS_50054.g2718**	13.82	unspecified product	No significant similarity found.
21	**EfaB_PLUS_23143.g1929**	13.76	PAN/Apple domain-containing protein	No significant similarity found.
22	**EfaB_MINUS_49504.g2799**	13.75	Glyceraldehyde 3-phosphate dehydrogenase NAD(P) binding domain, Glyceraldehyde 3-phosphate dehydrogenase catalytic domain-containing protein	*Eimeria maxima* glyceraldehyde-3-phosphate dehydrogenase, putative partial mRNA

23	**EfaB_MINUS_15576.g1334**	13.75	unspecified product	No significant similarity found.
24	**EfaB_MINUS_7742.g781**	13.74	Heat shock protein 70 family domain-containing protein	*Eimeria stiedai* heat shock protein 70 mRNA, complete cds
25	**EfaB_MINUS_3469.g353**	13.72	unspecified product	No significant similarity found.
26	**EfaB_PLUS_22450.g1860**	13.70	Heat shock protein 70 family domain-containing protein	*Eimeria tenella* uncharacterized protein (ETH_00000210), partial mRNA
27	**EfaB_MINUS_43533.g2732**	13.69	Alcohol dehydrogenase C-terminal, Beta-ketoacyl synthase N-terminal, AMP-dependent synthetase/ligase, Phosphopantetheine binding ACP domain, Acyl transferase, Beta-ketoacyl synthase C-terminal, Male sterility NAD-binding, Alcohol dehydrogenase N-terminal, Polyketide synthase ketoreductase domain, Polyketide synthase dehydratase domain, Ketoacyl-synthetase C-terminal extension domain-containing protein	*Eimeria tenella* Equisetin synthetase, related (ETH_00015480), partial mRNA
28	**EfaB_MINUS_26415.g2240**	13.66	Peptidase M1 membrane alanine aminopeptidase, Peptidase M1 alanyl aminopeptidase Ig-like fold, Peptidase M1 alanyl amino	MAG: *Rhodanobacteraceae bacterium* isolate bin_004 chromosome, complete genome
29	**EfaB_MINUS_22450.g1952**	13.53	unspecified product	No significant similarity found.
30	**EfaB_MINUS_16742.g1482**	13.46	Lactate/malate dehydrogenase N-terminal, Lactate/malate dehydrogenase C-terminal domain-containing protein	No significant similarity found.
31	**EfaB_PLUS_43143.g2627**	13.44	Glucose-methanol-choline oxidoreductase N-terminal, Glucose	No significant similarity found.

32	**EfaB_MINUS_13222.g1149**	13.42	unspecified product	No significant similarity found.
33	**EfaB_PLUS_2526.g312**	13.42	unspecified product	No significant similarity found.
34	**EfaB_MINUS_1001.g115**	13.40	unspecified product	No significant similarity found.
35	**EfaB_PLUS_3469.g348**	13.38	Tubulin/FtsZ GTPase domain, Tubulin/FtsZ 2-layer sandwich domain	*Toxoplasma gondii* cDNA, clone: XTG09340.2, full cDNA, XTG Sugano cDNA library
36	**EfaB_PLUS_42996.g2615**	13.37	unspecified product	No significant similarity found.
37	**EfaB_MINUS_15349.g1310**	13.33	FAS1 domain-containing protein	No significant similarity found.
38	**EfaB_PLUS_3469.g355**	13.28	MAM domain, Copper amine oxidase catalytic domain-containing protein	PREDICTED: *Cyclospora cayetanensis* uncharacterized LOC34622548 (LOC34622548), mRNA
39	**EfaB_PLUS_24701.g2017**	13.27	unspecified product	No significant similarity found.
40	**EfaB_MINUS_5504.g461**	13.25	Fructose-bisphosphate aldolase class-I domain-containing pr	PREDICTED: *Brassica napus* fructose-bisphosphate aldolase 8, cytosolic (LOC106367801), mRNA
41	**EfaB_MINUS_31498.g2397**	13.22	Enolase C-terminal TIM barrel domain, Enolase N-terminal d	*Cystoisospora suis* enolase (CSUI_007934), mRNA
42	**EfaB_rRNA_SSU_04**	13.17	unspecified product	*Eimeria falciformis* strain Chob2 18S small subunit ribosomal RNA gene, partial sequence
43	**EfaB_MINUS_13626.g1219**	13.04	unspecified product	No significant similarity found.
44	**EfaB_PLUS_9917.g892**	13.02	unspecified product	No significant similarity found.
45	**EfaB_PLUS_16835.g1438**	12.99	14-3-3 domain-containing protein	*Eimeria tenella* 14-3-3 protein mRNA, complete cds

46	**EfaB_MINUS_10843.g934**	12.99	unspecified product	*Kluyveromyces marxianus* strain CCT 7735 (UFV-3) chromosome 1 sequence
47	**EfaB_MINUS_32574.g2453**	12.98	Heat shock protein Hsp90 family domain-containing protein	*Eimeria acervulina* heat shock protein 90, putative partial mRNA
48	**EfaB_PLUS_22284.g1810**	12.88	Actin family domain-containing protein	*Eimeria tenella* clone Etm008A10 hypothetical protein mRNA, complete cds
49	**EfaB_MINUS_19717.g1715**	12.82	Glycosyl transferase family 35 domain-containing protein	*Eimeria maxima* glycogen phosphorylase family protein, putative partial mRNA
50	**EfaB_MINUS_43533.g2729**	12.82	unspecified product	No significant similarity found.
51	**EfaB_PLUS_13227.g1159**	12.81	Transcription factor GTP-binding domain, Elongation factor EF	PREDICTED: *Cyclospora cayetanensis* elongation factor 2 (LOC34620923), mRNA
52	**EfaB_MINUS_6710.g587**	12.79	A1 cistron-splicing factor AAR2, OST-HTH associated domain-containing protein	PREDICTED: *Cyclospora cayetanensis* uncharacterized LOC34619330 (LOC34619330), mRNA
53	**EfaB_PLUS_52892.g2747**	12.75	unspecified product	No significant similarity found.
54	**EfaB_PLUS_6743.g637**	12.74	unspecified product	No significant similarity found.
55	**EfaB_PLUS_15576.g1284**	12.72	unspecified product	No significant similarity found.
56	**EfaB_MINUS_3469.g352**	12.67	unspecified product	No significant similarity found.
57	**EfaB_PLUS_6035.g583**	12.64	Ribosomal protein L5 eukaryotic/L18 archaeal C-terminal, Ribosomal protein L5 euk	PREDICTED: *Amphibalanus amphitrite* 60S ribosomal protein L5-like (LOC122394442), mRNA
58	**EfaB_PLUS_2387.g274**	12.63	unspecified product	No significant similarity found.
59	**EfaB_MINUS_14978.g1271**	12.57	Ribosomal protein L3 domain-containing protein	*Eimeria mitis* 60S ribosomal protein L3, putative (EMH_0001660), partial mRNA
60	**EfaB_MINUS_56725.g2977**	12.57	Legume-like lectin domain-containing protein	No significant similarity found.

61	**EfaB_PLUS_13227.g1152**	12.52	Cyclophilin-type peptidyl-prolyl cis-trans isomerase domain-containing protein	*Eimeria tenella* 20 kDa cyclophilin precursor, putative (ETH_00011110), partial mRNA
62	**EfaB_MINUS_11030.g957**	12.48	Ribonucleotide reductase large subunit N-terminal, Ribonucleotide reductase la	PREDICTED: *Cyclospora cayetanensis* ribonucleoside-diphosphate reductase large subunit (LOC34622540), mRNA
63	**EfaB_PLUS_17195.g1490**	12.47	FAS1 domain-containing protein	No significant similarity found.
64	**EfaB_MINUS_34010.g2538**	12.44	unspecified product	*Eimeria acervulina* hypothetical protein, conserved partial mRNA
65	**EfaB_PLUS_49504.g2685**	12.38	Ribosomal protein L7Ae/L30e/S12e/Gadd45 domain-containing protein	PREDICTED: *Microtus oregoni* 60S ribosomal protein L7a (LOC121453412), mRNA
66	**EfaB_PLUS_7742.g732**	12.35	Citrate synthase, WD40 repeat domain-containing protein	No significant similarity found.
67	**EfaB_PLUS_7048.g660**	12.35	unspecified product	*Eimeria acervulina* hypothetical protein, conserved partial mRNA
68	**EfaB_PLUS_7742.g801**	12.34	unspecified product	No significant similarity found.
69	**EfaB_PLUS_10843.g913**	12.34	unspecified product	No significant similarity found.
70	**EfaB_PLUS_15724.g1357**	12.30	Phosphofructokinase domain-containing protein	*Eimeria acervulina* phosphofructokinase, putative partial mRNA
71	**EfaB_PLUS_800.g92**	12.27	Ribosomal protein S3Ae domain-containing protein	*Eimeria maxima* 40S ribosomal protein S3A, putative partial mRNA
72	**EfaB_PLUS_20371.g1665**	12.26	Ribosomal protein L4/L1e, Nascent polypeptide-associated complex NAC domain, 60S ribosomal protein L4 C-terminal domain-containing protein	*Polychytrium aggregatum* ribosomal protein L4 domain-containing protein (BJ171DRAFT_461012), mRNA
73	**EfaB_MINUS_13716.g1221**	12.25	Dehydrogenase E1 component domain-containing protein	No significant similarity found.

74	**EfaB_PLUS_21840.g1799**	12.24	DEAD/DEAH box helicase domain, Helicase C-terminal domain-containing protein	*Uncinocarpus reesii* 1704 eukaryotic initiation factor 4A-6 (UREG_07691), partial mRNA
75	**EfaB_PLUS_31498.g2291**	12.22	unspecified product	No significant similarity found.
76	**EfaB_PLUS_17907.g1564**	12.21	unspecified product	No significant similarity found.
77	**EfaB_PLUS_8675.g856**	12.20	Ribosomal protein S2 domain-containing protein	PREDICTED: *Cyclospora cayetanensis* 40S ribosomal protein SA (LOC34619493), mRNA
78	**EfaB_PLUS_7742.g792**	12.19	Ribosomal protein S5 N-terminal, Ribosomal protein S5 C-terminal domain-containi	PREDICTED: *Cyclospora cayetanensis* 40S ribosomal protein S2 (LOC34619846), mRNA
79	**EfaB_MINUS_20371.g1783**	12.19	Peptidase S8/S53 domain-containing protein	*Eimeria acervulina* Subtilase family protein, related partial mRNA
80	**EfaB_PLUS_7048.g687**	12.18	Ribosomal protein L10P, 60S ribosomal protein L10P insertion domain-containing pro	TPA_asm: *Neospora caninum* Liverpool, chromosome chrXII, complete genome
81	**EfaB_PLUS_17907.g1569**	12.15	Ribosomal protein S4e central region, RNA-binding S4 domain, Ribosomal protein	No significant similarity found.
82	**EfaB_PLUS_15349.g1250**	12.15	Starch synthase catalytic domain, AMP-activated protein kinase glycogen-bindin	*Eimeria tenella* uncharacterized protein (ETH_00004395), partial mRNA
83	**EfaB_PLUS_13227.g1156**	12.14	Pyruvate kinase barrel, Pyruvate kinase C-terminal domain-containing protein	*Eimeria tenella* pyruvate kinase (PYK) mRNA, complete cds
84	**EfaB_rRNA_LSU_02**	12.11	unspecified product	*Isospora sp.* RY-2015a 28S ribosomal RNA gene, partial sequence
85	**EfaB_MINUS_15724.g1412**	12.08	haloacid dehalogenase-like hydrolase domain-containing protein	No significant similarity found.
86	**EfaB_MINUS_13222.g1141**	12.07	Fatty acid desaturase domain, ELO family domain-containing protein	*Eimeria acervulina* fatty acyl-CoA desaturase, putative partial mRNA
87	**EfaB_PLUS_1048.g113**	12.05	unspecified product	No significant similarity found.

88	**EfaB_PLUS_1425.g181**	12.05	Ribosomal protein S4/S9 N-terminal, RNA-binding S4 domain-containing protein	PREDICTED: *Cyclospora cayetanensis* 40S ribosomal protein S9 (LOC34619750), mRNA
89	**EfaB_PLUS_8832.g871**	12.01	60s acidic ribosomal domain-containing protein	No significant similarity found.
90	**EfaB_MINUS_26136.g2217**	12.01	unspecified product	No significant similarity found.
91	**EfaB_MINUS_5792.g547**	12.00	Ribosomal Proteins L2 RNA binding domain, Ribosomal protein L2 C-terminal domain-containing protein	*Eimeria tenella* clone Etm077E01 hypothetical protein mRNA, complete cds
92	**EfaB_PLUS_20412.g1733**	11.98	unspecified product	No significant similarity found.
93	**EfaB_PLUS_50714.g2738**	11.97	Dynamin superfamily, Cation/H+ exchanger, Dynamin central domain, Dynamin G	*Eimeria maxima* dynamin-like protein, putative partial mRNA
94	**EfaB_MINUS_15648.g1392**	11.96	Ribosomal protein L13e domain-containing protein	No significant similarity found.
95	**EfaB_PLUS_1425.g183**	11.96	Notch domain, Thrombospondin type-1 (TSP1) repeat domain-containing protein	*Eimeria tenella* thrombospondin type 1 domain-containing protein, putative (ETH_00028220), partial mRNA
96	**EfaB_PLUS_9324.g881**	11.95	unspecified product	PREDICTED: *Drosophila montana* myb-like protein AA (LOC135427664), mRNA
97	**EfaB_PLUS_43143.g2628**	11.94	Glutamate/phenylalanine/leucine/valine dehydrogenase C-terminal, Glutamate/p	*Candidatus Sulfidibacterium* hydrothermale isolate EPR1 chromosome, complete genome
98	**EfaB_PLUS_15648.g1336**	11.93	unspecified product	No significant similarity found.
99	**EfaB_MINUS_7048.g692**	11.93	unspecified product	*Stutzerimonas stutzeri* strain KC chromosome, complete genome
100	**EfaB_PLUS_7048.g702**	11.92	Actin-depolymerising factor homology domain-containing protein	*Eimeria necatrix* actin depolymerizing factor, putative partial mRNA

Supplementary Table 4. Primer sequences used for qPCR analysis of Th1-related genes.

Name of Gene	Forward	Reverse
STAT1	GCCTCTCATTGTCACCGAAGAAC	TGGCTGACGTTGGAGATCACCA
Tbx21	CCACCTGTTGTGGTCCAAGTTC	CCACAAACATCCTGTAATGGCTTG
Il12b	TTGAACTGGCGTTGGAAGCACG	CCACCTGTGAGTTCTTCAAAGGC
Actin	CTACAATGAGCTGCGTGTG	TGGGGTGTTGAAGGTCTC
